# Anti-tumor Efficacy Assessment of the Sigma Receptor Pan Modulator RC-106. A Promising Therapeutic Tool for Pancreatic Cancer

**DOI:** 10.3389/fphar.2019.00490

**Published:** 2019-05-14

**Authors:** Anna Tesei, Michela Cortesi, Sara Pignatta, Chiara Arienti, Giulio Massimo Dondio, Chiara Bigogno, Alessio Malacrida, Mariarosaria Miloso, Cristina Meregalli, Alessia Chiorazzi, Valentina Carozzi, Guido Cavaletti, Marta Rui, Annamaria Marra, Daniela Rossi, Simona Collina

**Affiliations:** ^1^Biosciences Laboratory, Istituto Scientifico Romagnolo per lo Studio e la Cura dei Tumori (IRCCS), Meldola, Italy; ^2^Aphad S.r.l., Milan, Italy; ^3^Experimental Neurology Unit, School of Medicine and Surgery, Milan Center for Neuroscience, University of Milano-Bicocca, Monza, Italy; ^4^Department of Drug Sciences, Medicinal Chemistry and Pharmaceutical Technology Section, University of Pavia, Pavia, Italy

**Keywords:** Pancreatic cancer, pan-sigma receptor modulators, endoplasmic reticulum stress, unfolded protein response, proteasome inhibition

## Abstract

**Introduction:** Pancreatic cancer (PC) is one of the most lethal tumor worldwide, with no prognosis improvement over the past 20-years. The silent progressive nature of this neoplasia hampers the early diagnosis, and the surgical resection of the tumor, thus chemotherapy remains the only available therapeutic option. Sigma receptors (SRs) are a class of receptors proposed as new cancer therapeutic targets due to their over-expression in tumor cells and their involvement in cancer biology. The main localization of these receptors strongly suggests their potential role in ER unfolded protein response (ER-UPR), a condition frequently occurring in several pathological settings, including cancer. Our group has recently identified **RC-106**, a novel pan-SR modulator with good *in vitro* antiproliferative activities toward a panel of different cancer cell lines. In the present study, we investigated the *in vitro* properties and pharmacological profile of **RC-106** in PC cell lines with the aim to identify a potential lead candidate for the treatment of this tumor.

**Methods:** Pancreatic cancer cell lines Panc-1, Capan-1, and Capan-2 have been used in all experiments. S1R and TMEM97/S2R expression in PC cell lines was quantified by Real-Time qRT-PCR and Western Blot experiments. MTS assay was used to assess the antiproliferative effect of **RC-106**. The apoptotic properties of **RC-106** was evaluated by TUNEL and caspase activation assays. GRP78/BiP, ATF4, and CHOP was quantified to evaluate ER-UPR. Proteasome activity was investigated by a specific fluorescent-based assay. Scratch wound healing assay was used to asses **RC-106** effect on cell migration. In addition, we delineated the *in vivo* pharmacokinetic profile and pancreas distribution of **RC-106** in male CD-1 mice.

**Results:** Panc-1, Capan-1, and Capan-2 express both SRs. **RC-106** exerts an antiproliferative and pro-apoptotic effect in all examined cell lines. Cells exposure to **RC-106** induces the increase of the expression of ER-UPR related proteins, and the inhibition of proteasome activity. Moreover, **RC-106** is able to decrease PC cell lines motility. The *in vivo* results show that **RC-106** is more concentrated in pancreas than plasma.

**Conclusion:** Overall, our data evidenced that the pan-SR modulator **RC-106** is an optimal candidate for *in vivo* studies in animal models of PC.

## Introduction

Pancreatic cancer remains one of the most lethal tumor types for both men and women and, it represents the 11th most common cancer worldwide (Ilic and Ilic, 2016). WCRF reported that in 2018 there were 460,000 new cases, which mainly affected developed countries (Weledji et al., 2016). For this type of tumor, beneficial pharmaceutical approaches result challenging to develop, since the etiology as well as the triggering factors associated with PC remain undefined (Kim and Ahuja, 2015). Relying on the negative prognosis – the average 5-year survival rate is 6% or less (Siegel et al., 2014) – and on the lack of a concrete cure, PC urgently requires effective therapeutic strategies.

Over the past few decades, SRs, have been widely associated with aging- and mitochondria-associated disorders, such as Parkinson’s and Alzheimer’s disease and cancer (Martin et al., 1976; Su, 1982; Vaupel, 1983; Quirion et al., 1987; Maurice and Lockhart, 1997; Skuza, 2003; Peviani et al., 2014; Collina et al., 2017a,b). Moreover, although no endogenous SRs ligands have ever been found, and the specific role played by this orphan receptor family in cell biology has yet to be clarified, SRs are considered as potential therapeutic targets for neurodegenerative diseases and cancer. Accumulating evidence strongly suggests a pivotal role of these proteins in ER-UPR pathways, whose activation is frequently detected in many solid tumors (Shuda et al., 2003; Corazzari et al., 2017). In particular, the triggering of the UPR machinery in cancer is the result of neoplastic cells spreading in unfavorable environments characterized by hypoxia, low pH, high levels of ROS and inadequate glucose and amino acid supply, conditions that could compromise the correct ER protein folding. Under such stress conditions, SRs are activated to allow the cells survival, as broadly demonstrated by the direct involvement of S1R in UPR pathways (Hayashi, 2015; Penke et al., 2017). The decrease of Ca^++^ ion level in ER, the accumulation of misfolded or aggregated protein within the ER, the rise of ROS level due to stress conditions promote the exit of S1R from a dormant state and its activation as chaperon protein. Accordingly, the correct Ca^++^ signaling from the ER to the mitochondria, the transmission of the ER stress signal to the nucleus and the consequent increase of antistress and antioxidant proteins production are guaranteed (Hayashi and Su, 2007; Mori et al., 2013; Wang et al., 2015).

Only recently S2R has been cloned and its identity as TMEM97 has been postulated (Alon et al., 2017). TMEM97 is a transmembrane protein involved in cholesterol homeostasis, and its dysregulation has been associated to ER stress and to activation of the UPR, thus causing cellular lipid accumulation (Colgan et al., 2011). Notably, UPR is classically related to the maintenance of cellular homeostasis in secretory cells (i.e., pancreatic and immune cells), where the high demand for protein synthesis and secretion leads to proteostasis and cellular stress (Hetz, 2012; Moore and Hollien, 2012). Indeed, pancreatic cells have high hormone and enzyme secretory functions and possess highly developed ER. The role of ER stress in PC pathobiology and inflammation has been increasingly recognized as an important factor in tumorigenesis and chemoresistance (Yadav et al., 2014). Nonetheless, PC is extremely rich in stroma, is hypoxic and deficient in metabolites (Vasseur et al., 2010). A similar behavior can be found when cells grow under chronic metabolic stress conditions, favoring the activation of adaptive mechanisms, such as UPR and autophagy (Kondo et al., 2005; Moenner et al., 2007) the latter frequently associated to SR overexpression (Zeng et al., 2012; Mir et al., 2013). Altogether, these findings pointed out SRs as potential targets useful for inhibiting UPR machinery in PC.

Our research team is active in the SR modulation and recently we identified compound **RC-106** endowed with pan-SR modulatory activity (S1R antagonist and S2R agonist profile) and *in vitro* antiproliferative properties toward a panel of cancer cell lines (i.e., Capan-2, MDA-MB 231, PC3, and U87) (Rui et al., 2016; Rossi et al., 2017). These encouraging results led us to further investigate its potential in PC treatment. After preparing **RC-106** in a suitable amount to support the whole study, we deepened its antitumor properties and evaluated its capability to interfere with ER stress conditions. Lastly preliminary PK and biodistribution studies have been performed, to verify if **RC-106** is able to reach the target tissue.

## Materials and Methods

### RC-106 Synthesis

Reagents and solvents for synthesis were obtained from Sigma-Aldrich (Italy). Solvents were purified according to the guidelines in Purification of Laboratory Chemicals. Melting points were measured on SMP3 Stuart Scientific apparatus and are uncorrected. For FT-IR analysis a Spectrum One PerkinElmer spectrophotometer equipped with a MIRacle^TM^ ATR device was used. The IR spectra were scanned over wavenumber range of 4000–650 cm^-1^ with a resolution of 4 cm^-1^. Analytical thin-layer chromatography (TLC) was carried out on silica gel precoated glass backed plates (Fluka Kieselgel 60 F254, Merck); visualized by UV radiation, acidic ammonium molybdate (IV), or potassium permanganate. FC was performed with Silica Gel 60 (particle size 230e400 mesh, purchased from Merck). Proton NMR spectra were recorded on Bruker Avance 400 spectrometer operating at 400 MHz. ^13^C NMR spectra were recorded on 500 MHz spectrometer, operating at 125 MHz, with complete proton decoupling. UPLC-UV-ESI/MS analyses were carried out on a Acuity UPLC Waters LCQ FLEET system using an ESI source operating in positive ion mode, controlled by ACQUIDITY PDA and 4 MICRO (Waters). Analyses were run on a ACQUITY BEH C18 (50 mm × 2.1 mm, 1.7 mm) column, at room temperature, with gradient elution (solvent A: water containing 0.1% of formic acid; solvent B: methanol containing 0.1% of formic acid; gradient: 10% B in A to 100% B in 3 min, followed by isocratic elution 100% B for 1.5 min, return to the initial conditions in 0.2 min) at a flow rate of 0.5 mL min^-1^. Detailed synthetic procedure and characterization of intermediates and **RC-106** are reported in the Supplementary Material.

### Cell Cultures

#### 2D Cell Culture

Pancreatic adenocarcinoma Panc-1, Capan-1, and Capan-2, cell lines were purchased by the ATCC. All cell lines were grown in culture medium composed of DMEM/Ham’s F12 (1:1; Euroclone) supplemented with fetal calf serum (10%; Euroclone), glutamine (2 mM; Euroclone), and insulin (10 μg/mL; Sigma-Aldrich, St. Louis, MO, United States). All experiments were performed on cells in the exponential growth phase and checked periodically for mycoplasma contamination by MycoAlert^TM^ Mycoplasma Detection Kit (Lonza, Basel, Switzerland).

#### 3D-Cell Culture

Spheroids were obtained as previously described (Zanoni et al., 2016). Briefly, a rotatory cell culture system RCCS (Synthecon Inc., Houston, TX, United States) was used. The rotary systems were placed inside a humidified 37°C, 5% CO_2_ incubator and all procedures were performed in sterile conditions. Single cell suspensions of about 1 × 10^6^ cells/ml of Panc-1 were placed in the 50 mL rotating chamber at an initial speed of 12 rpm. Speed was increased as cells formed aggregates to avoid sedimentation. The culture medium was changed every 4 days and tumor spheroids with an equivalent diameter ranging from about 500–1300 μm were obtained in around 15 days. After formation, spheroids were transferred into a 96-well low-attachment culture plates (Corning Inc., Corning, NY, United States; one spheroid/well), containing 100 μL of fresh culture medium per well.

### Cell Viability Assays

#### MTS Assay

Cytotoxicity was assayed using CellTiter 96^®^ AQueous One Solution Cell Proliferation Assay (Promega, Milan, Italy). Cells were seeded onto a 96-well plate at a density of 3 × 10^3^ cells per well. Cell lines were exposed to increasing concentrations of the drug, ranging from 0.1 to 100 μM. The effect of the drug was evaluated after 24, 48, and 72 h of continued exposure. Two independent experiments were performed in octuplicate. The OD of treated and untreated cells was determined at a wavelength of 490 nm using a fluorescence plate reader.

Dose response curves were created by Excel software. IC_50_ values were determined graphically from the plot.

#### CellTiter-Glo^®^ 3D

Cell viability of Panc-1 spheroids was measured using a 3D cell viability assay (Promega, Milan, Italy). Briefly, homogeneous spheroids were removed from the 96-well low-attachment culture plate and placed separately in single wells of a 96-well opaque culture plate (BD Falcon). CellTiter-Glo^®^ 3D reagent was added to each well and the luminescence signal was read after 30 min with the GloMax^®^ bioluminescent reader (Promega).

#### Analysis of Morphological Parameters of 3D Tumor Spheroids

The analysis of morphological parameters were performed as previously described (Piccinini et al., 2017). Briefly, an inverted Olympus IX51 microscope (Olympus Corporation, Tokyo, Japan), equipped with a Nikon Digital Sight DS-Vi1 camera (CCD vision sensor, square pixels of 4.4 μm side length, 1600 × 1200 pixel resolution, 8-bit gray level; Nikon Instruments, Spa. Florence, Italy) was used to take images and for morphological analyses. The open-source ReViSM software tools was used to achieve morphological 3D, such as volume and sphericity, and to select morphologically homogeneous spheroids. For the experiments, Panc-1 spheroids characterized by spherical shape and by a diameter size ranging from 500 to 600 μM were selected.

### Real Time RT-PCR

Total cellular RNA was extracted using TRIzol reagent (Life technologies) in accordance with manufacturer’s instruction and quantified using the Nanodrop MD-1000 spectrophotometer system. Reverse transcription reactions were performed in 20 μL of nuclease free water containing 400 ng of total RNA using iScript cDNA Synthesis kit (Bio-Rad Laboratories, Hercules, CA). Real-Time PCR was run using 7500 Fast Real-Time PCR system (Applied Biosystems) and TaqMan assays to detect the expression of SIGMAR1, TMEM97, GRP78/BiP, ATF4, and CHOP genes.

Reactions were carried out in triplicate at a final volume of 20 μL containing 40 ng of cDNA template, TaqMan universal PCR Master Mix (2X), and selected TaqMan assays (20X). Samples were maintained at 50°C for 2 min, then at 95°C for 10 min followed by 40 amplification cycles at 95°C for 15 s, and at 60°C for 30 s.

The amount of mRNA was normalized to the endogenous genes GAPDH and HPRT-1.

### TUNEL Assay

TUNEL assay was performed as previously described (Tesei et al., 2007). Briefly, cells were fixed in 1% formaldehyde in PBS on ice for 15 min, suspended in 70% ice cold ethanol and stored overnight at 20°C. Cells were then washed twice in PBS and re-suspended in PBS containing 0.1% Triton X-100 for 5 min at 48°C. Thereafter, samples were incubated in 50 μL of solution containing TdT and FITC conjugated dUTP deoxynucleotides 1:1 (Roche Diagnostic GmbH, Mannheim, Germany) in a humidified atmosphere for 90 min at 37°C in the dark, washed in PBS, counterstained with propidium iodide (2.5 μg/mL, MP Biomedicals, Verona, Italy) and RNAse (10 kU/mL, Sigma–Aldrich) for 30 min at 48°C in the dark and analyzed by flow cytometry. Flow cytometric analysis was performed using a FACS Canto flow cytometer (Becton Dickinson, San Diego, CA, United States). Data acquisition and analysis were performed using FACSDiva software (Becton Dickinson). Samples were run in triplicate and 10,000 events were collected for each replicate.

### Western Blot

Western Blot were performed as previously described (Arienti et al., 2016). Briefly, Cell proteins were extracted with M-PER (Thermo Fisher Scientific) supplemented with Halt Protease Phosphatase Inhibitor Cocktail (Thermo Fisher Scientific).

Mini-PROTEANTGX^TM^ precast gels (4–20% and any kD; BIO-RAD) were run using Mini-PROTEAN Tetra electrophoresis cells and then electroblotted by Trans-Blot Turbo^TM^ Mini PVDF Transfer Packs (BIORAD). The unoccupied membrane sites were blocked with T-TBS 1X (Tween 0.1%) and 5% non-fat dry milk to prevent non-specific binding of antibodies and probed with specific primary antibodies overnight at 4°C. This was followed by incubation with the respective secondary antibodies. The antibody-antigen complexes were detected with Immun-Star^TM^ WesternC^TM^ kit (BIO-RAD).

The following primary antibodies were used: anti-sigma receptor (S18): sc-22948 (Santa Cruz Biotechnology inc.), anti-TMEM97, anti-caspase-3, and anti-caspase-9. Anti-vinculin (sc-5573) from Santa Cruz Biotechnology and anti-actin from Sigma Aldrich Inc., were used as housekeeping. Quantity One Software was used for analysis.

### Proteasome Activity Assay

Cells were seeded in 6-well plates at density of 250 × 10^3^ cells/well. Cells were treated with increasing concentration of **RC-106** and after 24 h total protein extracts were obtained: cells were washed 2 times with PBS and lysed with 100 μL of lysis buffer (Hepes 5 mM pH 7.5, NaCl 150 mM, Glycerol 10%, Triton X100 1%, MgCl2 1.5 mM, EGTA 5 mM). Protein concentration of samples was quantified using Bradford method. Proteasome activity was quantified as described below. Proteasome solution was composed by 40 μg of proteins, 10 μL of 10X proteasome buffer (Hepes pH 7.5 250 mM, EDTA pH 8.0 5 mM, NP-40 0.5%, SDS 0.01%) and 10 μL of 10 mM proteasome substrate (N-Succinyl-Leu-Leu-Val-Tyr-7-Amido-4-Methylcoumarin, 7.6 mg/mL; Sigma-Aldrich, United States). 100 μL of proteasome solution was loaded in wells of a black 96-well plate. The plate was then incubated at 37°C for 2 h and the fluorescence was measured in a microplate reader (excitation 380 nm, emission 460 nm; BMG-Labtech, Germany).

### Migration Scratch Wound Healing Assay

Cells were seeded in a 6-well plate and were incubated at 37°C until confluence of 90–100% was reached. Culture medium was then replaced by serum free medium. After 24 h, a scratch was made on cell monolayer using a plastic tip and wells were washed 2 times with PBS to remove detached cells and debris. Culture medium, with or without **RC-106**, was added to each well. Micrographs of the scratches were taken at 0 h, immediately after the scratch, and at 24, 48, and 72 h. Cell migration area was quantified using IMAGEJ software.

### Pharmacokinetic and Pancreas Distribution Studies

#### Animals and Biological Matrix Preparation

The experiments were performed in agreement with the Italian Law D. L.vo 4 marzo 2014, n. 26. The treatments involved male CD-1 mice and a unique number on the tail identified each animal. Mice were housed, in groups of four, in cages suitable for the species. After 5 days of adaptation to the local housing conditions, animals were housed in a single, exclusive, air-conditioned room to provide a minimum of 15 air changes/hour. The environmental controls were set to maintain the temperature at around 22°C and the relative humidity within the range 50 to 60%, along with an approximate 12:12 h light/dark cycle automatically controlled. Food (Mucedola Standard GLP diet) and water were available *ad libitum* throughout the entire duration of the study. All animals were weighted on the day of the treatment.

Mice (*n* = 4/time point) received an intraperitoneal administration (i.p., 10 mL/kg) of **RC-106** at 10 mg/kg. CD-1 male mice were exsanguinated under anesthesia (isoflurane) from the aorta at the following time points: 5, 10, 30, 120, 240, and 480 min. Blood samples were collected in tubes containing heparin, gently mixed and immediately placed on ice. Afterward, they have been centrifuged (3500 × *g*, at 4°C for 15 min), the obtained plasma has been collected and transferred to individually labeled tubes and frozen at -20°C until the analysis. Plasma samples were used for quantification of **RC-106**. Pancreas was taken by surgical resection after 20 min from the last treatment, washed in saline, dried on paper, weighted and frozen at -20°C. The organ was homogenized using a Velp OV5 homogenizer with 20 mM ammonium formiate buffer in a ratio of 1 g of tissue per 10 mL of buffer.

#### Sample Preparation

20 mg/mL stock solution (s.s.) of **RC-106** was prepared by dissolving the compound in DMSO. 1 mL of 5% Tween80 in H_2_O was slowly added to 500 μL of s.s. under stirring. Then 8.5 mL of water was gently spiked to obtain the 1 mg/mL formulation of **RC-106**.

Standard curves of **RC-106** were prepared for plasma and pancreas homogenate, and analyzed together with each QC and unknown sample set. For the PK and pancreas distribution sample analysis, plasma and pancreas homogenate samples (50 μL) were spiked in 200 μL of IS in MeOH (0.1 μg/mL of **RC-33**), followed by 2 min vortex mixing. Samples were centrifuged and transferred in UFLC vials. 5 μL aliquots of the collected samples were injected into the LC-MS/MS system. Standard calibration graphs were constructed by linear least-squares regression analysis on the analyte/IS area ratio plotted against sample concentration. Calibration ranges were from 5 to 1000 ng/mL for plasma, and from 5 to 500 ng/mL for pancreas homogenate. Accuracy values were determined in triplicates at three different concentrations (high, medium, and low) in the range of linearity of the calibration curves.

#### LC-MS/MS Conditions

Analyses were acquired on a Shimadzu AC20 UFLC system interfaced with an API 3200 Triple Quadrupole detector (AB Sciex). Data acquisition and control were performed using the Analyst^TM^ 6.1 (Applied Biosystems) Software. A Phenomenex Gemini-NX C18 (50 mm × 2 mm, 5 μm) column was selected to carry out the analytical evaluations. A gradient method was set up (Supplementary Table S1) and it provided the employment of water and methanol, both containing 0.1% of formic acid, at a flow rate of 0.3 mL/min. The LC eluate was directly introduced into the MS interface using the ESI in the positive ion mode. The MRM transitions m/z 181.2 were tracked (Supplementary Table S2).

## Results

### Chemistry

**Scheme 1:**
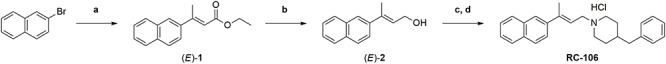
Synthesis of **RC-106**. Reagents and conditions: **(a)** (*E*)-ethyl crotonate, Pd EnCat^®^ 40, TEAC, NaOAc, DMF anhydrous, N_2_ atm., 105°C; **(b)** LiAlH_4_ (1M in THF), Et_2_O anhydrous, N_2_ atm., 0°C; **(c)** Ph_3_P, NBS, N_2_, -15/18°C; **(d)** 4-benzylpiperidine, Et_3_N, N_2_ atm., from -15/-18°C to r.t.

We studied an easy to handle synthetic route suitable to dispose of **RC-106** in a g-scale amount. The synthetic route is outlined in Scheme 1. Briefly, a Heck reaction between 4-bromobiphenyl and (*E*)-ethyl crotonate, using Palladium acetate microencapsulated in polyurea matrix (Pd EnCat^®^) as catalyst allowed to obtain the α,β-unsaturated ester (*E*)-**1** which was easily reduced to give allyl alcohol (*E*)-**2**, and then converted into **RC-106** according to Frøyen and Juvvik (1995). The use of Pd EnCat^®^ simplified the work-up procedure and more important avoided the heavy metal contamination of the product, which could compromise the *in vitro* and *in vivo* studies.

### Cell Biology

#### SRs Expression in Pancreatic Adenocarcinoma

We explored the expression of S1R and TMEM97/S2R genes in a panel of cell lines representative of pancreatic adenocarcinoma. The expression of S1R and TMEM97/S2R was evaluated in cells derived both from primary tumor and metastatic site (i.e., liver), characterized by different doubling time and different mutational status of p53, KRAS, P16/CDKN2A, and SMAD 4 (Table 1), the major driver-genes involved in the pathogenesis of PC (Sipos et al., 2003).

**Table 1:** Pancreatic ductal adenocarcinoma cell lines characterization.

	Site	Doubling time	p53	KRAS	P16/CDKN2A	SMAD 4
Panc-1	Primary tumor	52 h	Mut	Mut	Mut	WT
Capan-1	Liver metastasis	38 h	Mut	Mut	Mut	Mut
Capan-2	Primary tumor	96 h	WT	Mut	Mut	N.d

*All cell lines were purchased from ATCC*.

**Figure 1:**
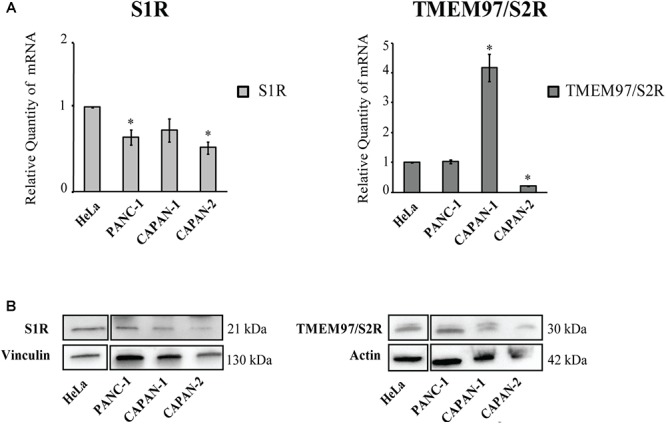
Relative Quantification (RQ) of the target genes Sigma 1 and TMEM97/S2R. **(A)** Analysis were performed with Real-Time PCR. RNA expression was normalized to GAPDH and HPRT-1. The RNA gene expression was relative to HeLa cell line (RQ = 1). Values are the mean ± SD of three independent experiments. ^∗^*P* < 0.05 vs. CTR. **(B)** Western Blot analysis of TMEM97 and S1R in PC cell lines. HeLa were used as reference sample. Images are representative of two independent experiments.

The expression level of SRs was determined by Real-Time qRT-PCR. We used cervix adenocarcinoma HeLa as reference sample, because of its high expression of both S1R and TMEM97/S2R (Bartz et al., 2009; Ebrahimi-Fakhari et al., 2015; Miki et al., 2015). All analyzed cell lines express SRs and no correlation between the tumor site and the expression level of both targets, as well as respect to the mutational status of p53 and KRAS was evidenced. In particular, S1R was expressed at similar levels in the PC cell lines. Conversely, differences about the expression of TMEM97/S2R have been evidenced in the three cell lines investigated, with the highest expression in Capan-1 (4-fold respect to the control line) and the lowest in Capan-2 cells (Figure 1). Basing on these results, we took into account the three cell lines to perform the biological evaluation.

#### *In vitro* Cytotoxic Activity

**Figure 2:**
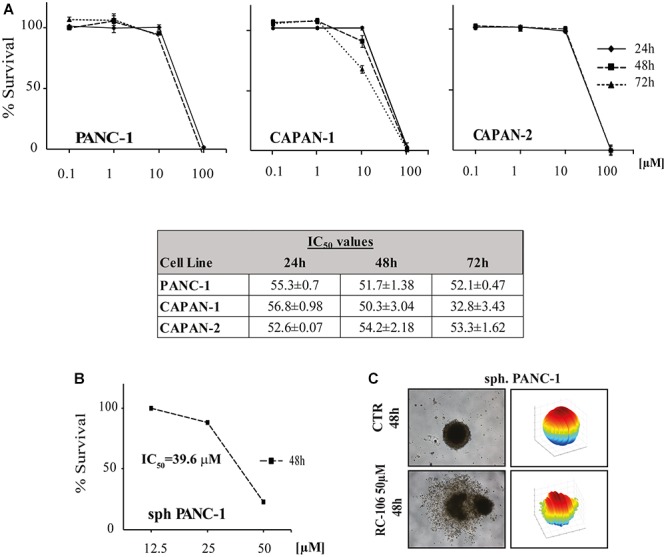
Cell viability of 2D and 3D cell lines. **(A)** *In vitro* cytotoxic activity of **RC-106** was evaluated in three PC cell lines. Cells were exposed to increasing concentration of the molecule for 24, 48, and 72 h. MTS assay was used to determine cell viability. Values are the mean ± SD of three independent experiments. **(B)** Homogeneous-size and shape pancreatic adenocarcinoma spheroids were treated with **RC-106** for 48 h at concentration ranging from 12.5 to 50 μM. Cell viability was measured using CellTiter-Glo 3D assay. **(C)** 3D spheroids shape reconstructed on representative brightfield images of Panc-1 spheroids treated with 50 μM of **RC-106** for 48 h. The corresponding 3D-shape of Panc-1 spheroids were obtained using ReViSM software tools.

We evaluated the *in vitro* cytotoxic activity of **RC-106** by MTS assay. Cells were treated for 24, 48, and 72 h with increasing concentrations, ranging from 0.1 to 100 μM. **RC-106** was effective in all cell lines tested independently from the exposure time (IC_50_ values ranging from 33 to 57 μM, Figure 2A). Encouraged by these results, we investigated the capability of **RC-106** to penetrate three dimensional structures mimicking tumor micronodules of about 500–600 μm in diameter. Panc-1 cells grown as 3D spheroids were treated with increasing concentrations of **RC-106** (12.5–50 μM for 48 h, Figure 2B). The results obtained with Panc-1 spheroids with a diameter up to 600 μm (IC_50_ = 39.55 μM, Figure 2B,C) are in line with those observed in 2D culture.

#### Pro-apoptotic Effect

**Figure 3:**
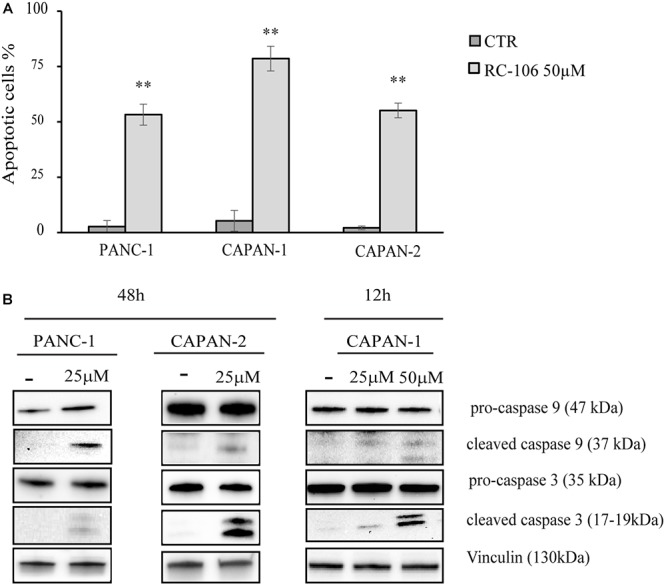
Apoptosis analysis. **(A)** TUNEL assay performed on Panc-1, Capan-1, and Capan-2 cell lines. Cells were treated with **RC-106** 50 μM for 48 h. Values are the mean ± SD of three independent experiments. ^∗∗^*P* < 0.01 vs. CTR. **(B)** Western Blot analysis of caspase 3 and 9 activation after 48 h treatment with **RC-106** 25 μM (Panc-1 and Capan-2) and after 12 h treatment with **RC-106** 50 μM (Capan-1). Images are representative of two independent experiments.

The apoptotic properties of **RC-106** was evaluated by TUNEL assay. The exposure time (48 h) and the drug concentration (50 μM) have been chosen according to the data resulting from cell viability assay. TUNEL assay showed a significant induction of apoptosis in treated samples compared to the untreated controls, with a percentage of apoptotic cells ranging from 53.25% ± 4.7 (Panc-1) to 78.55% ± 5.6 (Capan-1) (Figure 3A). Hence, we investigated the activation of caspase cascade by Western Blot analysis, treating cells with **RC-106** at different exposure times. We found that both caspases 3 and 9 were cleaved, in all cell lines after the treatment, indicating the activation of the intrinsic apoptotic pathway. To sum up, **RC-106** was able to activate both caspases in all the considered cell lines, but after different exposure times and concentrations. In detail, in Panc-1 and Capan-2 cell lines this event occurred after an exposure of 48 h to **RC-106** at 25 μM concentration, whereas in Capan-1 cell line after 12 h at 50 μM concentration (Figure 3B).

#### ER Stress and Unfolded Protein Response

**Figure 4:**
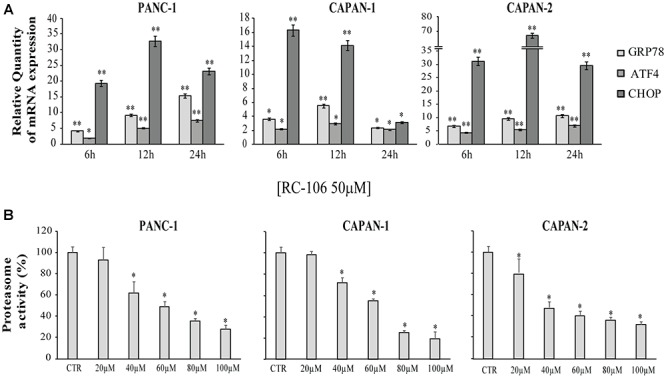
Relative Quantification (RQ) of the ER stress and UPR marker genes. **(A)** GRP78, ATF4, and CHOP mRNA expression levels were measured after a treatment with **RC-106** 50 μM for 6,12, and 24 h. Analysis were performed with Real-Time PCR. RNA expression was normalized to GAPDH and HPRT-1. In each time point tested the RNA gene expression was relative to the corresponding untreated control (RQ = 1). Values are the mean ± SD of three independent experiments. (^∗^*P* < 0.05 vs. CTR; ^∗∗^*P* < 0.01 vs. CTR). **(B)** Graphs represent the proteasome activity of PANC-1, Capan-1, and Capan-2, treated with increasing concentration of **RC-106** (20–100 μM) for 24 h. Data are expressed as the average percentage ± SD of at least three independent experiments and are compared to untreated controls (CTR 100%; ^∗^*P* < 0.05 vs. CTR).

The expression of the ER stress master proteins GRP78/BiP, ATF4, and CHOP, commonly used for the detection of UPR activation (Samali et al., 2010), was analyzed by Real-Time qRT-PCR. In general, the mRNA expression of all the investigated ER markers highly increased after the exposure to 50 μM of **RC-106**. In the two cell lines derived from primitive pancreatic tumor, Panc-1 and Capan-2, the trend is similar. In particular, GRP78/BiP and ATF4 mRNA levels increased after 24 h of treatment, while CHOP mRNA levels considerably increased after 12 h, then slightly declined after 24 h (Figure 4A). The highest increase in expression of CHOP was individuated in Capan-2 (70 fold higher than untreated cells). A different behavior was observed for the metastatic cell line Capan-1, where a faster switch-off of all ER markers was evidenced already starting from 12 h after the beginning of treatment.

All the cell lines were treated with increasing concentrations of **RC-106** (20–100 μM) to evaluate *in vitro* **RC-106** proteasome effect. After 24 h of treatment, **RC-106** was able to reduce proteasome activity in a dose dependent manner in all the PC investigated (Figure 4B). Capan-2 resulted the most sensible cell line as showed by the lowest concentration used to inhibit proteasome activity (20 μM). Instead the greatest proteasome inhibition is observed in Capan-1 cells but at highest concentration used (100 μM).

#### Cell Migration

**Figure 5:**
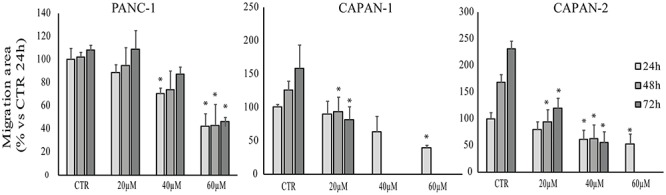
Effect of **RC-106** on Capan-1, Capan-2, and Panc-1 cell migration. Migration area of cells was quantified after 24, 48, and 72 h of treatment with increasing concentrations of **RC-106** (20–60 μM). Data are expressed as the average percentage ± SD of at least three independent experiments and are compared to controls (CTR, 100%; ^∗^*P* < 0.05 vs. respective CTR).

Scratch wound healing assay was performed to assess the effect of **RC-106** on cell migration. After the scratch, cells were treated with increasing concentration of **RC-106** (20–60 μM) and cell migration was evaluated after 24, 48, and 72 h. Capan-1 untreated cells migrated normally to refill the scratch present on cell monolayer. Cell migration was significantly reduced after 48 h of treatment with **RC-106** (*c* = 20 μM). Conversely, **RC-106** at concentrations of 40 and 60 μM reduced cell migration already after 24 h of treatment, whereas at major times these concentrations resulted too toxic, promoting cellular death. Capan-2 untreated cells migrated normally and continued to fill the empty space of the scratch for all considered times. **RC-106** 20 and 40 μM significatively reduceded Capan-2 cells migration ability after 48 and 72 h of treatment. **RC-106** 60 μM is too toxic and, as for Capan-1 cells, it was not possibile to quantify cell migration inhibition at 48 and 72 h. Panc-1 untreated cells migrated only for the first 24 h, then they slow down and stop migration. **RC-106** reduced Panc-1 cell migration in a dose dependent manner, but only in cells treated with 60 μM, migration was significatively reduced for all considered time points (Figure 5).

#### *In vivo* Pharmacokinetic and Pancreas Distribution Studies

**Figure 6:**
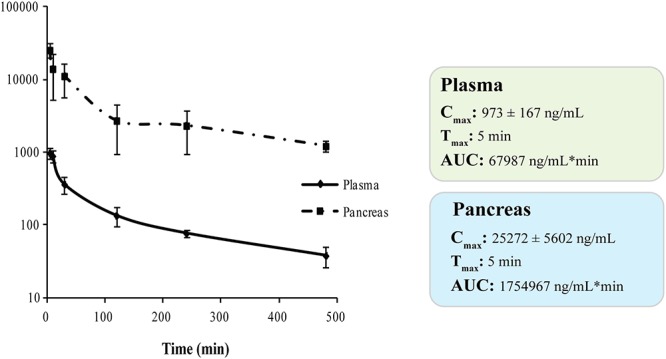
Plasma and pancreas PK parameters of **RC-106** after i.p., administration at 10 mg/kg.

We investigated the *in vivo* PK profile and pancreas distribution of **RC-106** in male CD-1 mice. Basing on our experience, we developed a rapid and sensitive UFLC-MS/MS method for detecting and quantifying **RC-106** in biological matrices (Rossi et al., 2013; Marra et al., 2016a,b). Briefly, chromatographic elutions were achieved on a reverse phase column and eluting under a gradient conditions (Supplementary Table S1). LC eluates were directly introduced into the MS interface using the ESI source and detected in positive ion mode (Supplementary Table S2). According to the structure of **RC-106**, parent ion m/z 356.5 and product ion m/z 181.2 – MRM transitions – were monitored during the analyses. Quantification of **RC-106** in plasma or pancreas homogenate were performed by generating 7 concentrations-calibration curves (5–1000 ng/mL for plasma, and 5–500 ng/mL for pancreas homogenate), employing **RC-33** as IS, 0.1 μg/mL in MeOH. Accordingly, concentrations of **RC-106** at each time point were extrapolated from the corresponding calibration curve. The developed method resulted suitable to separate **RC-106** from endogenous interferences. Afterward, CD-1 male mice received intraperitoneal administration at a concentration of 10 mg/kg. Plasma PK parameters are listed in Supplementary Table S3. **RC-106** showed a maximal concentration (Cmax) in plasma of 973.3 ng/mL (Tmax of 5 min) with an area under the curve (AUC_0-t_) of 67986.7 ng/mL^∗^min (Figure 6 and Supplementary Table S4). Interestingly, **RC-106** reached high concentrations also in pancreas with AUC_0-t_ of 1729315.7 ng/mL^∗^min, thus showing AUC_0-t_ pancreas/AUC_0-t_ plasma of about 25 times (Figure 6 and Supplementary Table S4).

## Discussion

Chemotherapy is the only therapeutic strategy effective in counteracting PC. Nevertheless, the pharmaceutical panorama counts very few effective molecules, since the etiology of this tumor is still elusive and specific therapeutic targets have not been identified yet. Recently, our research team highlighted that the PC cell lines express both S1R and S2R/TMEM97. Therefore, molecules acting *via* SRs pathway may play a positive role in counteracting PC. In the present work, we deepened the *in vitro* properties of the pan-SR modulator **RC-106** and evaluated its PK profile to define its potential as *lead* compound.

Pancreatic cancer cell lines Panc-1, Capan-1, and Capan-2, harboring a mutational status representative of clinical tumors and expressing both SRs, have been selected to delineate the *in vitro* **RC-106** profile, and used in all the experiments. The citoxicity tests clearly showed that **RC-106** exerts a strong antiproliferative and pro-apoptotic action in all considered cell lines, with IC_50_ values in the micromolar range. To straighten these data, we exploit the 3D cell culture spheroids, an *in vitro* model mimicking *in vivo* features, thus providing better read-outs for drug screening (Carragher et al., 2018). The analysis of 3D morphological parameters of Panc-1 cells, the only able to grow as 3D structure, showed a complete disaggregation of spheroid organization and cytoarchitecture, thus confirming both the strong cytotoxic activity of **RC-106** and its good penetration capability.

The cytotoxic activity of **RC-106** seems to be mostly attributable to the induction of the intrinsic apoptotic pathways. Herein, we focused on the failure of the adaptive response to restore protein-folding homeostasis. In fact, when UPR is inadequate to restore ER proteostasis, the pathway alternates its signaling toward a terminal UPR, leading to cellular death. To study the role of SRs in ER stress, we measured the expression of the key factors GRP78/BiP, ATF4, and CHOP. In detail, GRP78/BiP is one of the best characterized ER chaperones (Lee, 2005), whereas ATF4 and CHOP are both markers for the shift of the UPR signaling into the alternate signaling program called the “terminal UPR” (Oyadomari and Mori, 2004; Maly and Papa, 2014; Hetz and Papa, 2018).

The cellular exposure to **RC-106** induces a relevant increase of the considered key regulators of ER stress, being GRP78/BiP, ATF4, and CHOP overexpressed. To sum up, results of our experiments demonstrated that the antitumor activity of **RC-106** is related to the triggering of the “terminal UPR,” confirming the key role of SRs as ER Stress gatekeepers (Tesei et al., 2018). It is worth noting that some compounds able to activate the terminal “UPR” have already reached the clinic for the treatment of several neoplasia, including PC (Hetz et al., 2013; Wang et al., 2018). Among them, bortezomib an inhibitor of proteasome enzyme complex (Chen et al., 2011) deserve to be mentioned, even if its therapeutic use is hampered by its toxic side effects (Field-Smith et al., 2006; Chen et al., 2011; Kharel et al., 2018). Since previous works reported that the silencing or the presence of loss-of-function mutations of S1R lead to an imbalance of protein degradation (Fukunaga et al., 2015; Dreser et al., 2017; Kim, 2017), we extent the evaluation to proteasome inhibition activity. **RC-106** resulted able to inhibit the proteasome activity in all the examined cell lines in a dose dependent manner. As a last step of cell biology investigation, we performed the scratch wound healing assay suitable for estimating the local spreading of cancer cells in the tissues/organs. The results showed that **RC-106** is able to decrease PC cell motility in a dose dependent manner, suggesting its therapeutic efficacy also in advanced disease.

Taken together the aforementioned results suggest **RC-106** as a valuable candidate for the treatment of PC. Considering that tissue distribution in target organ is at the core of drug discovery and development process, having a direct impact on pharmacology, we conclude our study performing PK and pancreas distribution evaluations. The results show that **RC-106** is 25 times more concentrated in pancreas than plasma, reaching a concentration similar or even higher (C_max_ about 70 μM) than those required to be effective in all the *in vitro* experiments considered in this work.

## Conclusion

Pancreatic cancer treatment is one of the most relevant challenges that the scientific community will have to face in the 21st century. Although novel approaches for PC have been recently proposed, chemotherapy still remains the only effective option to mitigate and counteract the devastating outcome. We herein propose **RC-106**, a pan-SR modulator with S1R antagonist and S2R agonist profile discovered by our research team, as a valuable compound for *in vivo* investigation. Obtained results clearly demonstrated that it is effective against PC, *via* apoptotic pathways, driven by both SR modulation and proteasome complex inhibition. We also deepen the mechanism of action, studying the role played by SR as ER gatekeepers. The so-obtained results demonstrated that **RC-106** is able to modulate UPR in response to ER stress, enhancing the expression of GRP78/BiP, ATF4, and CHOP. Furthermore, **RC-106** affected not only the viability of PC lines, but also their metastatic potential. Not last in importance, our lead compound it is able to reach the target tissue.

In conclusion, basing on pharmacological and PK profile we suggest the pan-SR modulator **RC-106**, as an optimal candidate for proof of concept *in vivo* studies in animal models of PC.

## Ethics Statement

Autorizzazione ministeriale 433/2016-PR: the procedures were authorized by the national authority (Istituto Superiore di Sanità, authorization number 433/2016-PR) and adhered to all the applicable institutional and governmental guidelines for the treatment of laboratory animals (Italian D.L.vo n. 26/2014).

## Author Contributions

AT and SC: conceptualization. AT, MM, GD, DR, and SC: experimental design and methodology. MC, SP, CA, AlM, CM, AC, VC, MR, and AnM: investigation. MC, AlM, MR, and AnM: writing-original draft preparation. AT, MC, GD, MM, MR, and SC: writing-review and editing. AT, GC, and SC: supervision. AT and SC: project administration. All authors contributed to manuscript revision, and read and approved the submitted version.

## Conflict of Interest Statement

The authors declare that the research was conducted in the absence of any commercial or financial relationships that could be construed as a potential conflict of interest.

## Abbreviations

ATCC: American Type Culture Collection
ATF4: activating transcription factor 4
CHOP: C/EBP homologous protein
CTR: control
DMSO: dimethyl sulfoxide
ER: endoplasmic reticulum
ESI: electrospray ionization
FC: flash chromatography
FITC: fluorescein isothiocyanate
GAPDH: glyceraldehyde 3-phosphate dehydrogenase
GRP78: 78-kDa glucose regulated protein
HPRT-1: hypoxanthine phosphoribosyltransferase 1
IS: internal standard
LC: liquid chromatography
MAM: mitochondria associated ER membrane
M-PER: Mammalian Protein Extraction Reagent
MRM: multiple reaction monitoring
MS: mass spectrometry
NMR: nuclear magnetic resonance
OD: optical density
PBS: phosphate buffered saline
PC: Pancreatic cancer
PK: pharmacokinetic
QC: quality control
RCCS: rotary cell culture system
ROS: reactive oxygen species
S1R: sigma 1 receptor
S2R: sigma 2 receptor
SD: standard deviation
SRs: sigma receptors
TdT: terminal deoxynucleotidyl transferase
TLC: thin layer chromatography
TMEM97: transmembrane protein 97
UFLC: ultra-fast liquid chromatography
UPLC: ultra performance liquid chromatography
UPR: unfolded protein response
UV: ultraviolet
WCRF: World Cancer Research Fund.
