# Supplementary material for: Anti-tumor Efficacy Assessment of the Sigma Receptor Pan Modulator RC-106. A Promising Therapeutic Tool for Pancreatic Cancer
Source: Front Pharmacol. 2019 May 14;10:490. doi: 10.3389/fphar.2019.00490 (PMC6530361; doi:10.3389/fphar.2019.00490)
Supplement: Supplementary file 1 [file Table_1.DOCX]

**SUPPLEMENTARY MATERIAL**

**Preclinical anti-tumor efficacy assessment of the Sigma Receptor Pan modulator RC-106. A new therapeutic tool for pancreatic cancer.**

Anna Tesei*^#1^, Michela Cortesi^1#^, Sara Pignatta^1^, Chiara Arienti^1^, Giulio Massimo Dondio^2^, Chiara Bigogno^2^, Alessio Malacrida^3^, Mariarosaria Miloso ^3^, Cristina Meregalli^3^, Alessia Chiorazzi^3^, Valentina Carozzi^3^, Guido Cavaletti^3^, Marta Rui^4^, Annamaria Marra^4^, Daniela Rossi^4^ and Simona Collina* ^4^

1 Biosciences Laboratory, Istituto Scientifico Romagnolo per lo Studio e la Cura dei Tumori (IRCCS), Meldola, Italy

2 Aphad S.r.l., Via della Resistenza 65, 20090 Buccinasco Milan-I, Italy

3 Experimental Neurology Unit, School of Medicine and Surgery& Milan Center for Neuroscience, University of Milan Bicocca, Via Cadore 48, 20900 Monza-I, Italy

4 Medicinal Chemistry and Pharmaceutical Technology Section, Department of Drug Sciences, University of Pavia, Pavia,Italy.

*corresponding author

#the authors contributed equally to this work

**Table of contents**

| 1. Synthesis of RC-106  2. Experimental procedures | S2  S3 |
| --- | --- |
| 2.1. (*E*)-ethyl 3-(naphthalen-2-yl)but-2-enoate [(*E*)-**1**] | S3 |
| 2.2. (*E*)-3-(naphthalen-2-yl)but-2-en-1-ol [(*E*)-**2**] | S4 |
| 2.3. (*E*)-4-benzyl-1-(3-(naphthalen-2-yl)but-2-en-1-yl)piperidine [**RC-106**] | S4 |
| 3. Pharmacokinetic and pancreas distribution studies, LC-MS/MS conditions | S5 |

**1.** **Synthesis of RC-106**

Compound (*E*)-**1** was obtained *via* a Heck reaction, modifying a procedure already set-up by our group. In detail, an in depth analysis of several synthetic protocols, involving conventional and unconventional methods, led us to identify the optimal conditions, which envisage the use of Palladium acetate microencapsulated in polyurea matrix (Pd EnCat® 40) instead of naked palladium acetate. The encapsulation in this porous vehicle minimizes the exposure of palladium to the air, simplifies the work-up procedure and avoids the heavy metal contamination of the product. Accordingly, the catalyst was added to a solid mixture of 2-bromonaphthalene, tetraethylammonium chloride and sodium acetate, under nitrogen atmosphere. Before suspending the solid mixture in dimethylfomamide, ethyl crotonate was added and lastly, the temperature was increased up to 105 °C. The purification of the crude (*E*)-**1**, through an initial liquid extraction followed by a flash chromatography, gave access to the desired (*E*)-**1** as a single stereoisomer. The α,β-unsatured ester (*E*)-**1** was converted into the allylalcohol (*E*)-**2**, using a LiAlH_4_-mediated reduction, under nitrogen atmosphere. After quenching with a solution of saturated ammonium chloride, the crude (*E*)-**2** was subjected to a liquid extraction and a flash chromatography, which led to the pure compound (*E*)-**2**. RC-106 was obtained by adopting a one-pot synthesis of tertiary amines from alcohols and amines *via* alkoxyphosphonium salts, a procedure described by the pioneers Frøyen P. and Juvvik P.. However, some modifications (*i.e.* reagent equivalents, the temperature and the timing of reagent additions) to the initial protocol were required to improve the yield of the reaction. Therefore, *N*-Bromosuccinimide was added to a solution of (*E*)-**2** and triphenylphosphine in anhydrous tetrahydrofuran, under nitrogen atmosphere, at -15/-18 °C. Once the intermediate alkoxy phosphonium was generated a solution of 4-benzylpiperidine and triethylamine in tetrahydrofuran was added dropwise. One hour later the reaction reached the completeness and the crude **RC-106** was purified by performing a liquid extraction and a flash chromatography, which gave access to the desired product, as confirmed by NMR, UPLC-MS and IR analyses. Then, it was converted in the corresponding hydrochloride.

**2. Experimental procedures**

Proton chemical shifts (d) are reported in ppm with the solvent reference relative to tetramethylsilane (TMS) employed as the internal standard (CDCl_3_, δ = 7.26 ppm). The following abbreviations are used to describe spin multiplicity: s = singlet, d = doublet, t = triplet, q = quartet, m = multiplet, br = broad signal, dd = doublet-doublet, td = triplet-doublet. The coupling constant values are reported in Hz. Carbon chemical shifts (d) are reported in ppm relative to TMS with the respective solvent resonance as the internal standard (CDCl_3_, δ = 77.23 ppm).

**2.1. (*E*)-ethyl 3-(naphthalen-2-yl)but-2-enoate [(*E*)-1]**

Under nitrogen atmosphere, 2-bromonaphthalene (1.00 g, 4.80 mmol, 1.0 equiv.), anhydrous sodium acetate (NaOAc) (0.79 g, 9.70 mmol, 2.0 equiv.), tetraethylammonium chloride (TEAC) (1.60 g, 9.70 mmol, 2.0 equiv.) and Pd EnCat® 40 (loading 0.4 mmol/g, 0.12 g, 0.048 mmol, 0.01 equiv.) were introduced in a dried round flask. To the solid mixture, 14 mL of anhydrous dimethylformamide (DMF) and 0.90 mL of (*E*)-ethyl crotonate (7.24 mmol, 1.5 equiv.) were added, and the resulting suspension was stirred, at 105 °C. After three hours the reaction took on black coloring. The suspension was filtered through a pleated filter and the obtaioned solution was extracted with Et_2_O (3 x 25 mL) and washed with brine (3 x 25 mL), The organic phase was dried (anhydrous Na_2_SO_4_), filtered and, after removal of the solvent under reduced pressure. The crude was purified by silica gel flash chromatography (*n*-hexane/ethyl acetate 97:3, v/v), yielding 0.62 g of compound (*E*)-**1**.

Yield: 58%; white solid; mp: 49-50 °C; IR (cm^-1^): 2391, 2337, 2286, 2143, 1704, 1540, 1413, 1129; ^1^H NMR (400 MHz) in CDCl_3_, δ (ppm) 7.91 – 7.82 (m, 4H, Ar), 7.63 (dd, *J* = 8.6 and 1.7 Hz, 1H, Ar), 7.56 – 7.50 (m, 2H, Ar), 6.33 (d, *J* = 0.9 Hz, 1H, CH_3_CC*H*), 4.28 (q, *J* = 7.1 Hz, 2H, COOC*H_2_*CH_3_), 2.73 (d, *J* = 0.9 Hz, 3H, C*H_3_*CCH), 1.37 (t, *J* = 7.1 Hz, 2H, COOC*H_2_*CH_3_).

**2.2. (*E*)-3-(naphthalen-2-yl)but-2-en-1-ol [(*E*)-2]**

Under nitrogen atmosphere, lithium alluminium hydride (LiAlH_4_ 1M in THF, 8.32 mL, 8.32 mmol, 1 equiv.) was added dropwise to a solution of α,β-unsaturated ester (*E*)-**1** (2.0 g, 8.32 mmol, 1 equiv.) in anhydrous diethylether (Et_2_O) (20 mL) cooled to 0 °C. After stirring the mixture for 15 minutes, 10 mL of ethyl acetate (AcOEt) and 10 mL of a saturated solution of ammonium chloride (NH_4_Cl) were added. The phases were separated and the organic phase was dried over anhydrous Na_2_SO_4_ and concentrated under reduced pressure. The obtained crude was purified by silica gel flash chromatography (gradient mobile phase, from *n*-hexane/AcOEt, 70:30, v/v to *n*-hexane/AcOEt, 40:60, v/v). 1.35 g of compound (*E*)-**2** were obtained.

Yield: 82%, white solid; mp: 81-82°C; IR (cm^-1^): 3647, 3339, 2985, 2372, 2217, 1747, 1705, 1670, 1596, 1540, 1506, 1375, 1099, 1008; ^1^H NMR (400 MHz) in CDCl_3_, δ (ppm): 7.93 – 7.78 (m, 1H, Ar), 7.63 (d, *J* = 8.6 Hz, 1H, Ar), 7.50 – 7.43 (m, 1H, Ar), 6.17 (t, *J* = 6.6 Hz, 1H, CH_3_CC*H*), 4.46 (d, *J* = 6.6 Hz, 2H, CCHC*H_2_*), 2.22 (s, 3H, C*H_3_*CCH), 1.60 (s, 1H, OH).

**2.3. (*E*)-4-benzyl-1-(3-(naphthalen-2-yl)but-2-en-1-yl)piperidine [RC-106]**

Under nitrogen atmosphere, six different portions of *N*-Bromosuccinimide (NBS, 6 x 0.16 g, 5.30 mmol, 1.4 equiv.) were added to a -15/-18° C cooled solution of allylalcohol (*E*)-**2** (0.75 g, 3.78 mmol, 1.0 equiv.) and triphenylphosphine (PPh3, 1.49 g, 5.68 mmol, 1.5 equiv.) in anhydrous THF (13 mL). After 30 minutes, a solution of 4-benzylpiperidine (0.80 mL, 4.54 mmol, 1.2 equiv.) and triethylamine (Et_3_N, 1.06 mL, 7.57 mmol, 2.0 equiv.) in 2 mL of THF was introduced into the reaction mixture and then the reaction was slowly allowed to reach room temperature. The stirring was continued for 1 additional hour. Afterwards the solution was transferred into a funnel and subjected to a liquid extraction with 50 mL of a saturated solution of sodium carbonate (Na_2_CO_3_) in H_2_O and Et_2_O (3 x 50 mL). The organic phase was dried over anhydrous Na_2_SO_4_ and concentrated under reduced pressure. The obtained crude was purified by silica gel flash chromatography (mobile phase AcOEt/ *n*-hexane, 80:20, v/v). 0.88 g of RC-106 were obtained.

Yield: 65%; yellow solid; mp: 221-222°C; IR (cm^-1^): 3049-2977, 2926, 2848, 2514, 1597, 1482, 1453-1434, 1287-1157, 1039, 940, 895, 855, 819, 744, 689; ^1^H NMR (400 MHz) in CDCl_3_, δ (ppm): 7.86 – 7.76 (m, 4H, Ar), 7.62 (dd, *J* = 8.6 and 1.6 Hz, 1H, Ar), 7.51 – 7.41 (m, 2H, Ar), 7.33 – 7.26 (m, 2H, Ar), 7.22 (d, *J* = 7.3 Hz, 1H, Ar), 7.17 (d, *J* = 7.1 Hz, 2H, Ar), 6.10 (t, *J* = 6.5 Hz, 1H, CH_3_CC*H*), 3.25 (s, 2H, NC*H_2_*), 3.05 (s, 2H, NC*H_2_*), 2.58 (d, *J* = 6.9 Hz, 2H, CHC*H_2_*N), 2.18 (s, 3H, C*H_3_*CCH), 2.04 (d, 2H, CHC*H_2_*Ph), 1.70 (s, 1H, CHC*H_2_*Ph), 1.59 (s, 2H, NCH_2_C*H_2_*), 1.42 (s, 2H, NCH_2_C*H_2_*); ^13^C NMR (500 MHz) in CDCl3, δ (ppm): 140.7, 140.4, 136.9, 133.4, 132.5, 129.1, 128.1, 128.0, 127.6, 127.5, 126.0, 125.7, 125.6, 124.2, 124.1, 57.2, 54.1, 43.2, 37.9, 32.2, 16.1; UHPLC-ESI-MS: tR = 2.20 minutes, >98% pure (λ 245 nm), *m/z* = 356 [M + H]^+^.

**3. Pharmacokinetic and pancreas distribution studies, LC-MS/MS conditions**

**Table S1.** Gradient program for bioanalysis of **RC-106** employing a UFLC Shimadzu system coupled with API 3200 (AB Sciex) and a Phenomenex Gemini-NX C18 column.

| **Flow (mL/min)** | **Injection**  **Volume (µL)** | **Time (min)** | **% H_2_O^*^** | **% MeOH^*^** |
| --- | --- | --- | --- | --- |
| 0.3 | 5 | 0.01 | 98 | 2 |
|  |  | 0.05 | 98 | 2 |
|  |  | 2.5 | 2 | 98 |
|  |  | 3.5 | 2 | 98 |
|  |  | 4 | 98 | 2 |
|  |  | 5 | 98 | 2 |

^*^ Both mobile phases were added to 0.1% formic acid.

**Table S2.** MS/MS parameters. DP: Declustering Potential, EP: Entrance Potential, CE: Collision Energy, CXP: Collision cell Exit Potential. MS parameters were as follows: Temperature: 450 °C, Gas: 40, Gas2: 40, CUR: 40, IS: 5500.

|  | **RC-106** |
| --- | --- |
| Parent ion (m/z) | 365.5 |
| Product ion (m/z)^a^ | 181.2 |
| Product ion (m/z) | 166.3 |
| DP (V) | 26 |
| EP (V) | 6 |
| CE (V) | 27 |
| CXP (V) | 1 |

^a^ Transition used for quantification.

**Table S3.** Plasma and pancreas concentration vs time profiles of **RC-106** after i.p. administration at 10 mg/kg in mice (n=4 for time point).

| **Time (min)** | **plasma** | | **pancreas** | |
| --- | --- | --- | --- | --- |
|  | C (ng/mL) | SD | C (ng/g) | SD |
| **5** | 973 | 167 | 25272 | 5602 |
| **10** | 884 | 169 | 13944 | 8706 |
| **30** | 361 | 98 | 11084 | 5332 |
| **120** | 134 | 40 | 2709 | 1781 |
| **240** | 76 | 8 | 2306 | 1369 |
| **480** | 38 | 12 | 995 | 962 |

Concentration values are means ± SD of 4 mice for time point.

**Table S4.** Plasma and pancreas PK parameters of **RC106** after if administration at 10mg/kg

| **Pharmacokinetic parameters^*^** | **Plasma** | **Pancreas** |
| --- | --- | --- |
| **AUC_0-t_ (ng/mL*min)** | 67987 | 1729316 |
| **t_1/2_ (min)** | 202 | 240 |
| **C_max_ (ng/mL) ± SD^#^** | 973 ± 167 | 25272 ± 5602 |
| **T_max_ (min)** | 5 | 5 |

^*^Pharmacokinetic parameters calculated on the mean tissue concentration vs time.

**^#^** Maximum concentration value is mean ± SD of 4 mice at the T_max_.
